# Natural Repellents as a Method of Preventing Ant Damage to Microirrigation Systems

**DOI:** 10.3390/insects13040395

**Published:** 2022-04-18

**Authors:** Luis de Pedro, Juan Antonio Sanchez

**Affiliations:** Department of Crop Protection, Biological Control and Ecosystem Services, Instituto Murciano de Investigación y Desarrollo Agrario y Medioambiental, C/Mayor s/n, La Alberca, 30150 Murcia, Spain; juana.sanchez23@carm.es

**Keywords:** ants, microirrigation systems, subsurface drip irrigation, repellents, olfactometer, p-anisaldehyde, cinnamon essential oil, ethyl anthranilate

## Abstract

**Simple Summary:**

Ants play an essential role in most agroecosystems. However, these insects can occasionally be detrimental to agricultural microirrigation equipment by chewing on tubing parts and causing uneven water distribution along the crops. One of the alternatives traditionally proposed to avoid this damage is the incorporation of substances into the tube material that deter or reduce chewing activity. However, the few attempts made prior to this research were unsuccessful due to the unsuitability of the selected substances and the methods used to integrate them into the tubing. In this study, we assessed the protective efficacy of this method in pipes used for subsurface drip irrigation (SDI). Unlike traditional attempts, we selected nontoxic compounds previously proven to be repellent against ants and integrated them into drip tubing through complex processes such as plastic compounding, injection and extrusion. The use of this type of tubing in a crop where significant ant damage is often reported revealed minimum damage when compared to control tubing containing no repellent additives. This suggests the efficiency of this method in providing protection against ants, but further studies are recommended prior to the commercialization of the designed system.

**Abstract:**

Ants are important because they damage agricultural equipment, including microirrigation systems. The aim of this research was to assess the efficiency of the incorporation of repellents in drip irrigation tubing as a method of protection against ant damage. Unlike previous studies, we tested a series of nontoxic compounds that are repellent to ants. First, we assessed their repellent effects on a local ant species via olfactometer trials. Then, the candidates showing the best results (cinnamon essential oil, p-anisaldehyde and ethyl anthranilate) were incorporated via compounding, injection and extrusion to polyethylene tubing to test their efficiency in the field. Field tests showed high damage levels in the control tubing containing no repellents, presumably caused by up to six different ant species (*Cardiocondyla batesii*, *Plagiolepis pygmaea*, *P. schmitzii*, *Solenopsis* sp., *Tapinoma nigerrimum* and *Tetramorium semilaeve*). In contrast, the pipes containing the three selected compounds remained almost intact, with the treatment including ethyl anthranilate showing no damage at all. These results suggest the strong repellent potential of the selected compounds, even when integrated into plastic, as well as the apparent success of the proposed methodology against the damage caused by ants. The diversity of damage-causing agents that exist in or above the soil strongly encourages further studies to determine the overall efficiency of repellents in protecting irrigation pipes.

## 1. Introduction

Ants (Hymenoptera: Formicidae) are one of the most successful and influential groups of organisms on the planet [1]. They occur in large numbers in many different types of habitats and ecosystems, and their ecological importance in most of them is immeasurable [1,2]. In agriculture, the role of ants has always been controversial, with substantial evidence of both beneficial and harmful effects on crops. Among the negative effects, one of the most widely studied is the disruption of the biological control of honeydew-producing pests, to whom ants often provide protection in exchange for a sugar supply [3,4,5]. However, the overall impact of these associations seems to depend on the ecological context, and many studies have reported positive effects on biological control [6,7,8,9]. Much clearer causes of ant damage in agricultural crops are, for example, the seed predation observed in different species of harvesting ants [10] or the defoliation caused by leaf-cutting ants (Formicidae: Myrmicinae: Attini) in neotropical agroecosystems [11,12]. 

A lesser-known adverse effect of ants on crops is the damage they can cause to agricultural materials [13] and, more specifically, to different types of microirrigation systems. The first reports on this type of damage date back to the 1970s, when several studies [14,15,16] conducted on Hawaiian crops demonstrated the ability of ants to harm drip irrigation systems. These authors observed uneven water distribution along their experimental crops and realized that the orifices of many emitters of the installed polyethylene surface (SD) and subsurface drip irrigation (SDI) pipes were exceptionally enlarged. Their analyses attributed this damage to the activity of several ant species, such as *Pheidole megacephala* (F.), *Solenopsis geminata* (F.) and *Linepithema humile* (Mayr). Drip irrigation damage caused by ants has rarely been reported in the scientific literature. The only recent mentions were made by Stansly and Pitts [17], who summarized the previous findings in Hawaii, and Boman [18]. In a grove in Florida, the latter observed modern pressure compensating drip tubing showing emitters with significant portions missing and ant body parts inside. The undesired effects of ants on microsprinkler irrigation systems have also been occasionally reported, including emitter clogging [19,20,21] or impairment of the silicon diaphragms of micropulsators [22]. 

Ants still represent a major problem for microirrigation systems in certain agricultural areas, as reflected by the frequent cases of growers reporting failures that were presumably due to ant activity. In recent years, many growers from different areas of southeastern Spain found clear evidence of leakages in emitters of the SDI systems installed in their crops, coinciding with areas of intense ant activity at both ground and soil levels. Several samples of dysfunctional polyethylene tubes were sent to the Laboratory of Biological Pest Control and Ecosystem Services of the IMIDA (Instituto Murciano de Investigación y Desarrollo Agrario y Medioambiental, Murcia, Spain) in 2018 and 2019 to determine the cause of the failures. Significant structural damage, as well as the presence of ants or ant body parts, were observed in many emitters, revealing that ant damage to SDI systems is recurrent in this area. Microirrigation and, specifically, SDI have been progressively gaining recognition in arid and semi-arid regions, such as the Mediterranean Basin, where water saving is a primary issue. In Spain, for example, more than 3 million ha are irrigated each year, and drip irrigation accounts for more than 40% of agricultural water use and for up to 80% in some Mediterranean provinces [23,24]. Therefore, ant damage could represent a serious threat to the economies of these areas, and finding solutions to this problem has become a matter of critical importance. 

Different alternatives have traditionally been considered to minimize the damage and costs associated with ant activity in microirrigation systems. Indirect damage, such as emitter clogging, seems to be easily manageable, for example, by increasing orifice diameters [21]. Direct damage has been more difficult to control. Several authors have proposed the development of tube and orifice configurations that impose physical barriers to prevent the entry of ants into both SDI and microsplinker systems [18,22,25,26]. This strategy showed promising results in preliminary trials with ants [22,25] but is not expected to achieve satisfactory control of other larger agents that can damage irrigation structures, such as birds (in surface systems) or rodents [17,27,28]. Another alternative is the incorporation of repellent substances into irrigation systems with the aim of deterring or reducing chewing activity [16,17]. Unlike physical barriers, repellents are expected to protect against agents of different sizes if those agents are sensitive to such substances. To date, most attempts in this regard have focused on the incorporation of chemicals into the irrigation water in drip systems, with poor results due to the toxicity of the selected products and their low persistence in the environment (T. Munuera, personal communication). More persistent protection could be achieved by incorporating these substances into the tubing structure. However, this strategy has been practically neglected, with only one study including ants as target organisms [16] and a few more against rodents [29,30]. These studies evidenced the same toxicity and low persistence problems mentioned above. For example, in [16], repellents were randomly selected and incorporated just by soaking the tube in different solutions of the tested compounds, which resulted in poor protection and toxicity to ants. Regarding rodents, Sorensen et al. [29] only sprayed drip tubing with a commercial animal repellent (Ropel^®^ (MCB LLC, Boca Raton, FL, USA)), which was not effective in controlling damage. This suggests that the poor performance could be attributed more to failures in the methodology employed than to the strategy itself.

Under these circumstances, in the present research, we aimed to determine whether the incorporation of repellents into microirrigation tubing may be a suitable strategy to avoid the damage caused by ants. Unlike previous attempts, our proposal involves the use of natural and nontoxic substances previously reported as potential ant repellents and their incorporation into polyethylene SDI tubing by complex processes such as extrusion and injection. In the first phase of our research, the repellent effect against the ants of a series of selected candidate substances was assessed through olfactory assays. In the second phase, we tested the protective efficiency of the tubing material containing the most repellent compounds in the field. 

## 2. Materials and Methods

### 2.1. Repellents

The selection of the substances to be tested as repellents in olfactory and field trials was made through an extensive literature search. The selected compounds had to meet the following requirements: (1) show previous repellent effects on one or more ant species; (2) be natural compounds that are nontoxic to the environment and humans at low doses; (3) show physicochemical properties that allow their injection and extrusion in polyethylene pipes; and (4) be commercially available and have an affordable cost that allows the acquisition and use of reasonable amounts of the product for marketing purposes. We identified five compounds fulfilling these conditions: ethyl anthranilate, eucalyptol (1,8-cineole), p-anisaldehyde (4-methoxybenzaldehyde), cinnamon essential oil and citronella essential oil [31,32,33,34,35,36,37,38,39]. Permethrin was also tested because it is a plant-derived and biodegradable compound, although it is synthetic [40,41,42]. Physicochemical properties of each compound, including toxicity, were mainly checked at “PubChem” (https://pubchem.ncbi.nlm.nih.gov/) (accessed on 1 May 2019). Further information on these substances is provided in the Supplementary Materials (Table S1). Hereafter, they are referred to as EA (ethyl anthranilate), EUC (eucalyptol), ANI (p-anisaldehyde), CEO (cinnamon essential oil), CIT (citronella essential oil) and PER (permethrin). CEO was obtained from Idai Nature S.L. (La Pobla de Vallbona, Valencia, Spain), while EA, EUC, ANI, CIT and PER were supplied by Ernesto Ventós S.A. (Sant Just Desvern, Barcelona, Spain).

### 2.2. Experimental Design

#### 2.2.1. Olfactory Testing

Prior to the experiments, an ant colony was established in 2018 in the facilities of the IMIDA. This colony was established with specimens of the species *Pheidole pallidula* (Nylander) (Hymenoptera: Formicidae). This species was chosen because of its high abundance in the soil of Mediterranean crops [43], including Andalusian olive groves [44] similar to the experimental crop where field trials were later conducted (See Section 2.2.2.). Specimens were obtained from an ant nest taken from the base of a lemon tree in the village of Santo Ángel (Murcia, Spain). Since then, laboratory rearing has been maintained (rearing conditions: 25 ± 2 °C, 65 ± 10% relative humidity (RH) and 16:8 (L:D) photoperiod) using water, honeydew and house crickets (*Acheta domesticus* L. (Orthoptera: Gryllidae)) as nutritional sources. 

**Figure 1:**
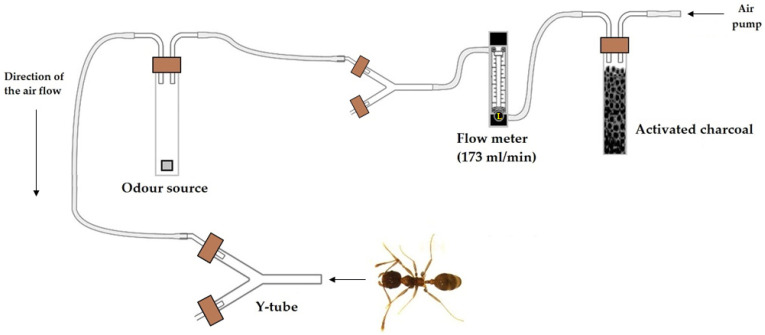
Schematic diagram of the Y-tube olfactometer and experimental setup. This figure is a modification of “Figure 3”, included in the manuscript authored by Saad et al. [46] (doi: 10.1038/srep13697) and licensed under CC BY 4.0 (http://creativecommons.org/licenses/by/4.0/).

The ants’ response to the different compounds was assessed using a Y-tube olfactometer in a series of trials performed under controlled conditions (25 ± 2 °C, 65 ± 10% RH, 2516 lux). The olfactometer followed the model described by Akol et al. [45] and Saad et al. [46], with minor modifications in the size of the Y-tube glass. In our study, the Y-tube was 0.5 cm in diameter, with a 6 cm base and two 5 cm arms. Odor sources were placed inside two 125 mL crystal jars, one connected to each arm of the olfactometer with Teflon tubes (0.5 cm in diameter). Air was circulated through the system by an air pump, which pumped the air first through an active charcoal filter for purification, then through a flowmeter (Supelco, Bellefonte, Pennsylvania, USA) which channeled the air at 173 mL/min. Airflow was established based on previous experiments with similar-sized insects [47,48,49,50]. Air was ejected from the flowmeter and split into two streams by Y-tube glass, with each arm connected with one of the crystal jars. Thus, air passed through the odor source and then into the two arms of the olfactometer. The system is graphically represented in Figure 1.

For all olfactory tests, similar-sized *P. pallidula* workers from the laboratory colony were used. Each specimen was tested individually by placing it at the base of the Y-tube using a soft paint brush and a 3 min response period. A response was considered positive if the individual walked at least 4 cm into one of the arms and was scored according to the chosen arm/odor source. If an individual did not make a choice within the 3 min period, it was considered a negative response and was discarded from subsequent analyses. This procedure was repeated until 40 positive responses were recorded for each comparison between odor sources. To minimize any spatial effect on the choice, the Y-olfactometer arms were flipped 180° every 5 tested individuals. In addition, for every 10 tested individuals, the Y-tube was replaced by a new one, and jars were switched. After every 20 individuals tested, Y-tubes and jars were rinsed with soap, water and alcohol and replaced with new ones.

In the first set of trials, we tested the repellence of the natural compounds selected in the previous stage in liquid form. To this end, a small piece of filter paper (1 × 1 cm^2^) was introduced into each crystal jar. One of the pieces was impregnated with 2 µL of the selected compound and the other was not, constituting the odorless control. The repellence of these compounds was also pairwise compared to establish a hierarchy among them. In a second set of trials, we repeated the same methodology using 1 × 1 × 0.2 cm^3^ samples of low-density polyethylene (LDPE) containing 5% of the compounds that showed the highest repellence as odor sources in the previous set. LDPE fragments that did not contain those compounds were used as controls. Finally, the last set of trials consisted of the same types of assays, but used high-density polyethylene (HDPE) samples with two concentrations (3% and 5%) of the same compounds. Plastic samples were produced and provided by AIMPLAS (Instituto Tecnológico del Plástico, Paterna, Valencia, Spain).

#### 2.2.2. Field Trials 

The three best-performing compounds in the olfactory trials were incorporated into HDPE drip tubing (16 mm Ø, wall thickness: 1.1 mm; Sistemas AZUD, Murcia, Spain) at a 5% concentration. The density of plastic and concentration of substances were established based on the results of the previous experiment. Masterbatches of these compounds at 5% were obtained by compounding performed by AIMPLAS, then injected into the emitters and extruded to the external layer of the pipe by AZUD. The emitters of the experimental tubing were integrated inside the tubing at a spacing of 0.5 m. These emitters were pressure compensated using a passageway and a silicon diaphragm that adjusted to pressure changes, and their discharge rate was established at 2.3 L/h. Moreover, a 0.5 mm Ø orifice was artificially drilled in the center of the diaphragm to ease the ants’ access and the identification of their potential damage.

**Figure 2:**
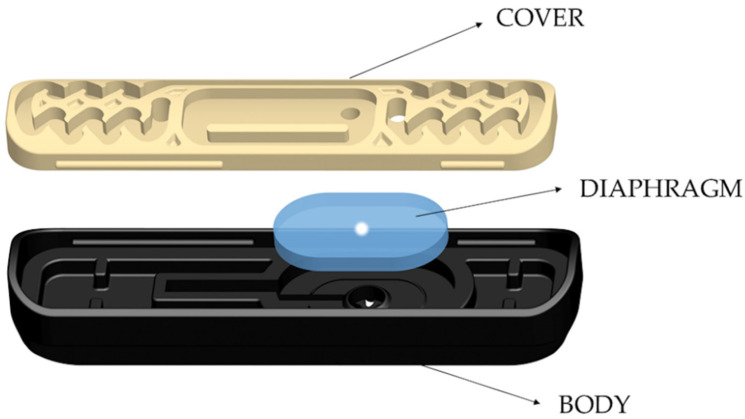
Diagram of an emitter showing its three components: body, silicon diaphragm and cover. The water flows from the body to the cover, passing through the diaphragm. Illustration provided by Teresa Munuera (Sistemas AZUD).

**Figure 3:**
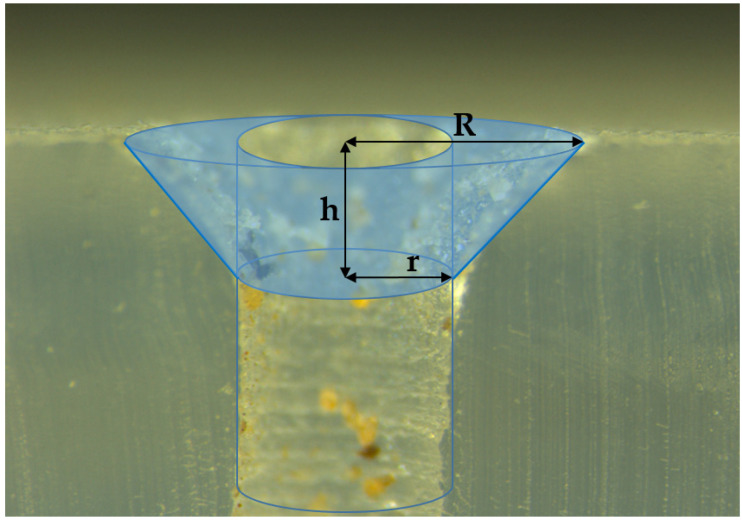
Transversal cut of a damaged silicon diaphragm showing the “truncated cone” caused by ant activity; R: radius of the top surface of the cone; h: height; r: radius of the base (i.e., of the inner orifice of the diaphragm). The volume of the diaphragm eroded by ants is represented in translucent blue.

Different fragments of this tubing were buried in the field on 15 July 2020, imitating an SDI installation and exposed to ant activity to test the effectiveness of the proposed method against ants. The experimental field was a 4.63 ha olive orchard located near the locality of Albendín (Córdoba Province, 37° 41′ 68” N 04° 05′ 08.9” W) where, in 2018 and 2019, ant damage was reported by the grower. The effect of the incorporation of repellents on the ability of ants to cause damage was tested in a randomized, block-design experiment with three replications of four treatments (i.e., the three compounds and a tubing control containing no repellent). A smaller area of the crop (approximately 2.6 ha), where ant activity and damage was previously observed, was selected for the trials and divided into three blocks, each containing four lines of trees and four alleys between the trees (width: **8** m). In each alley of each block, an isolated 10 m fragment of tubing containing one of the compounds (or no compound in the control) was buried approximately 0.5 m under the ground. Moreover, to increase the foraging activity of the ants, the tubes were filled with commercial honeydew (Biobest Sistemas Biológicos, Almería, Spain). The materials were exposed to ant activity for 3 months (July–October 2020). After this exposure time, the tubes were recovered and taken to the IMIDA facilities to be examined. Ten emitters were randomly selected and cut from each fragment. Each emitter was disassembled, and each component (body, silicon diaphragm and cover) (Figure 2) was examined for the presence of ants or signs of damage. A stereomicroscope system (Leica DFC490, Leica Microsystems, Wetzlar, Germany) was used for examination, image recording and damage quantification. Ants were identified at the species level following the keys of Martínez et al. [51] and Lebas et al. [52]. The number of ants and the volume of diaphragm eroded were assessed in each emitter. Ants eroded the silicon diaphragm on the sides of the artificial orifice, producing a characteristic “truncated cone” (Figure 3). The damage intensity was estimated as the volume of this cone, excluding the cylinder of the inner orifice itself and was calculated using the following expression:$\;V=\left(\frac{\pi h}{3}\right)\left(R^{2}+r^{2}+R.r\right)-\left(\pi hr^{2}\right)\;$(Figure 3). 

### 2.3. Data Analyses

In olfactory tests, differences in choice between each odor source were tested by chi-square goodness-of-fit tests using the “chisq.test” function in the R “stats” package [53]. In field trials, generalized linear mixed models (GLMM) were used to test for the effect of the repellent treatment on the number of ants in the emitters. GLMM were run using the function “glmmPQL” (MASS package) [54] set to the negative binomial distribution to account for the overdispersion of the data [53]. The type of repellent was introduced in the models as a fixed factor and the block and emitter as random factors. However, generalized linear models (GLM), run with the function “glm” (“stats” package) set for normally distributed data (i.e., family = Gaussian), were used to test for the effect of the type of repellent on the proportion of diaphragms damaged and volume of diaphragm eroded. The χ^2^ and p-values for the fixed factor were obtained from the Wald test using the “ANOVA” function in the R “car” package [53]. The post hoc, pairwise multiple comparisons among the treatments were run using Tukey’s test with the function “glht” in the “multcomp” package in R [55]. 

## 3. Results

### 3.1. Olfactory Testing

**Figure 4:**
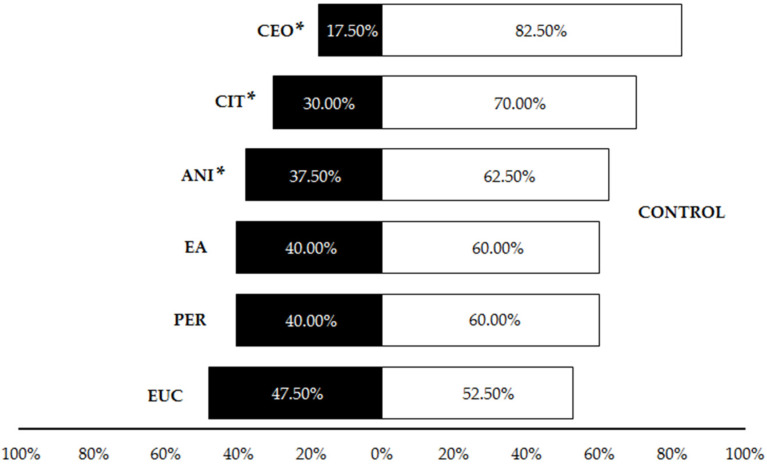
Percentage of *Pheidole pallidula* workers responding to each compound when offered simultaneously an odorless control in the olfactory tests. Asterisks (*) indicate significant differences between compounds and controls in each choice (*p* ≤ 0.05). CEO: cinnamon essential oil; CIT: citronella essential oil; ANI: p-anisaldehyde; EA: ethyl anthranilate; PER: permethrin; EUC: eucalyptol.

**Figure 5:**
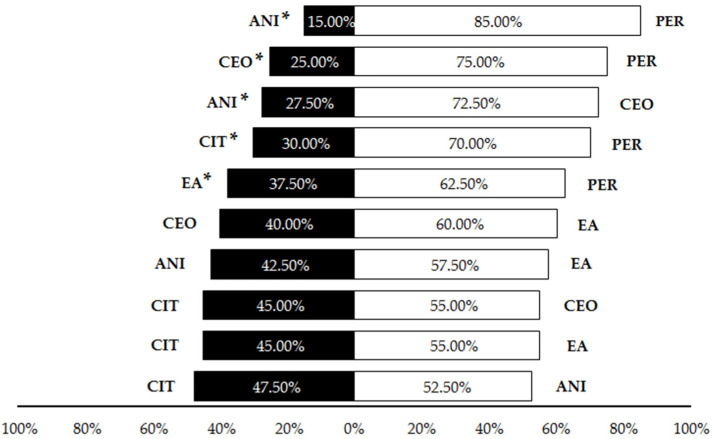
Percentage of *Pheidole pallidula* workers responding to each odor source in different paired combinations of compounds in the olfactory tests. Asterisks (*) indicate significant differences between compounds in each choice (*p* ≤ 0.05). CEO: cinnamon essential oil; CIT: citronella essential oil; ANI: p-anisaldehyde; EA: ethyl anthranilate; PER: permethrin.

The first set of olfactory tests revealed that cinnamon essential oil, citronella essential oil and p-anisaldehyde were significantly repellent in liquid form to *P. pallidula* in comparison to the control, with a significantly higher proportion of ants choosing the olfactometer arm without any repellent (CEO: 82.5%; CIT: 70%; ANI: 62.5%) (Table 1, Figure 4). Conversely, controls were not significantly preferred by *P. pallidula* when permethrin, ethyl anthranilate (60% of choices) and eucalyptol (52.5%) were offered as odor sources (Table 1, Figure 4). Due to its low repellence, eucalyptol was discarded from subsequent trials. In the pairwise comparisons among the other five compounds, a significantly higher proportion of ants chose the arm with permethrin than with the other repellents (Table 1, Figure 5) and thus this compound was discarded from the trials with plastic. Additionally, a significant repellent effect was observed in the comparison ‘CEO vs. ANI’, with a strong repellence by the latter revealed by the higher proportion of choices (72.5%) for cinnamon oil (Table 1, Figure 5). The other pairwise comparisons showed similar proportions of choices for both compounds (Table 1, Figure 5). 

**Figure 6:**
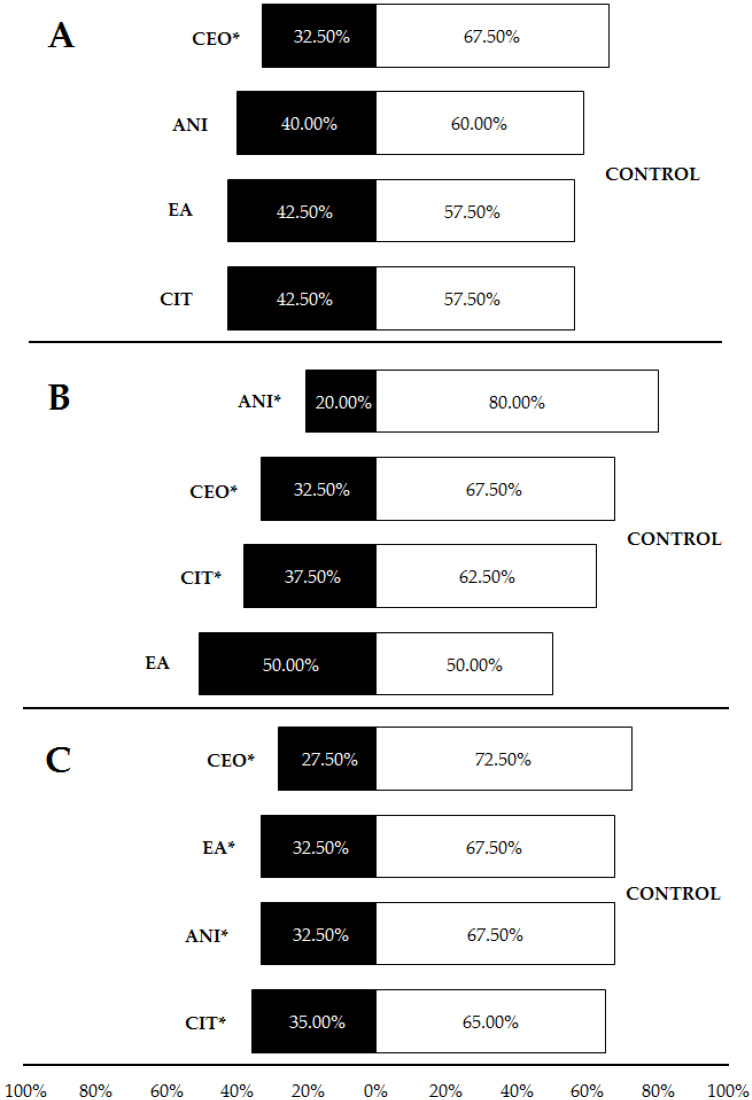
Percentages of *Pheidole pallidula* workers responding to each compound incorporated into plastic when offered simultaneously an odorless control (also in plastic) in the olfactory tests. In (**A**), compounds were incorporated into LDPE plastic at a concentration of 5%; in (**B**) and (**C**), compounds were incorporated into HDPE plastic at a concentration of 3% (**B**) and 5% (**C**). Asterisks (*) indicate significant differences between compounds and controls in each choice (*p* ≤ 0.05). CEO: cinnamon essential oil; CIT: citronella essential oil; ANI: p-anisaldehyde; EA: ethyl anthranilate.

When repellents were incorporated into LDPE plastic, comparisons with controls showed that cinnamon oil was significantly repellent (67.5% of choices for controls), unlike p-anisaldehyde (60%), ethyl anthranilate and citronella essential oil (57.5%) (Table 1, Figure 6A). When incorporated into HDPE at 3%, p-anisaldehyde, cinnamon essential oil and citronella essential oil were also repellent, leading to a proportion of 80, 67.5 and 62.5% of choices for controls, respectively. Ethyl anthranilate showed no repellence (50% of responses for each source) under these circumstances (Table 1, Figure 6B). Finally, the trials with HDPE samples at 5% concentration revealed significant repellence for all tested compounds (CEO: 72.5% of choices for controls; EA and ANI: 67.5%; CIT: 65%) (Table 1, Figure 6C). These results suggest a certain positive effect of both plastic density and compound concentration on the repellent effect of ants. 

### 3.2. Field Trials

Based on the results of the olfactory tests, CEO, EA and ANI were selected for field testing and injected into and extruded to the experimental tubing of HDPE at 5%. 

**Figure 7:**
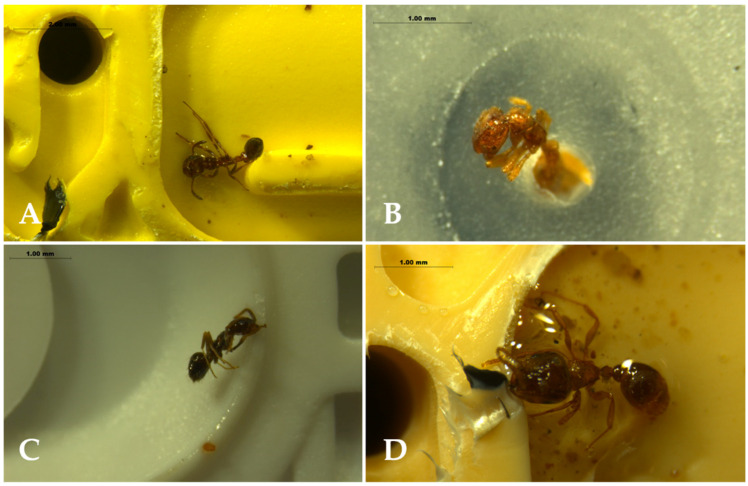
Some examples of ant specimens detected and photographed inside the emitters; (**A**): one specimen of *Cardiocondyla batesii* located under the outer surface (treatment: ANI); (**B**): one individual of *Solenopsis* sp. stuck in the inner orifice of the diaphragm (treatment: CEO); (**C**): a specimen of *Plagiolepis schmitzii* in the inner chamber that surrounds the diaphragm (treatment: EA); (**D**): *Tetramorium semilaeve* under the outer surface of a control tube.

Ants were the only potential agents observed inside the emitters, and six ant species were identified: *Cardiocondyla batesii* Forel, *Plagiolepis pygmaea* (Latreille), *Plagiolepis schmitzii* Forel, *Solenopsis* sp., *Tapinoma nigerrimum* Nylander and *Tetramorium semilaeve* André (Figure 7). Some specimens were not complete and could not be reliably identified due to their poor condition. *Solenopsis* sp. was the most abundant species inside the emitters (56% of the ants observed), followed by *Pl. pygmaea* (16.8%), *T. nigerrimum* (11.2%), *Pl. schmitzii* (8.8%), *C. batesii* (4.8%) and *T. semilaeve* (2.4%). 

**Figure 8:**
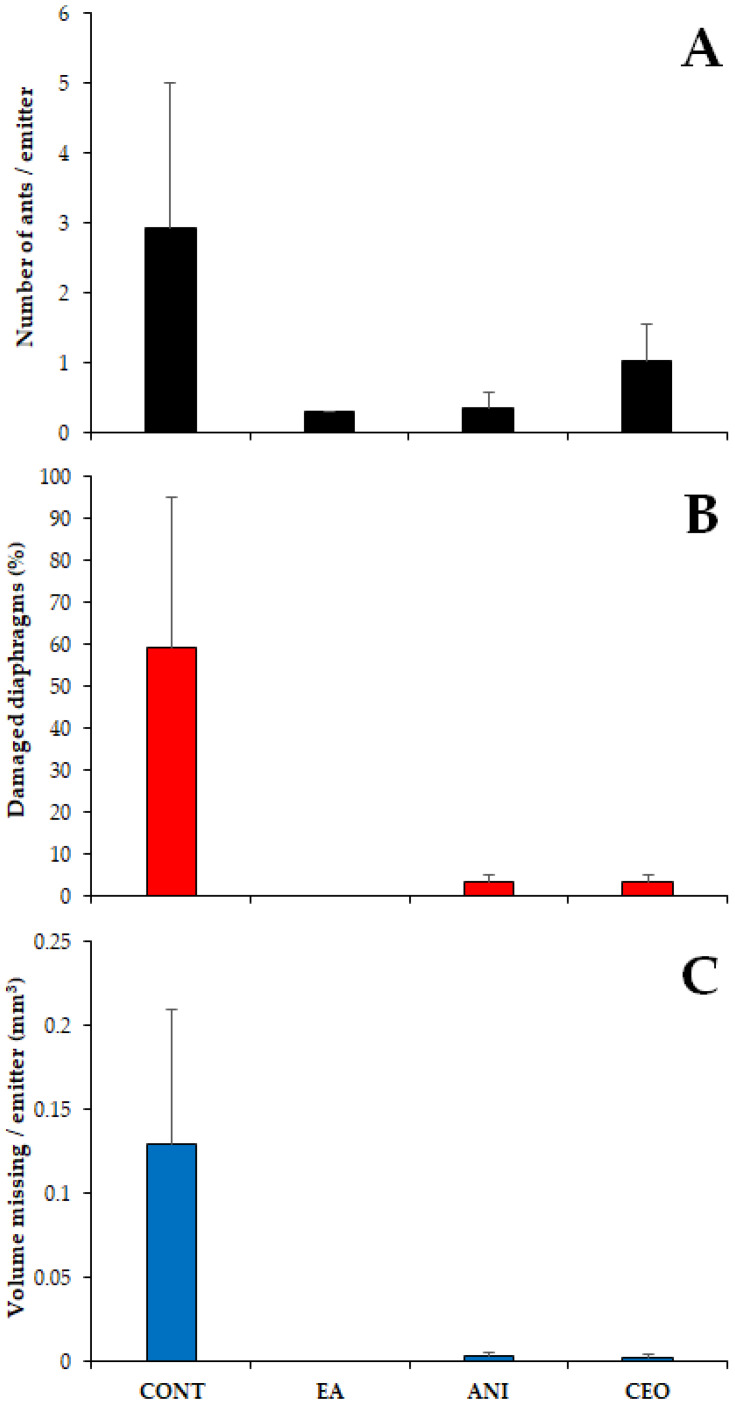
Number of ants (**A**), percentage of emitters showing diaphragm damage (**B**) and volume of diaphragm eroded (**C**) (mean ± SE) observed in the different treatments. CONT: control. EA: ethyl anthranilate. ANI: p-anisaldehyde. CEO: cinnamon essential oil.

The mean number of ants per emitter was significantly affected by the repellent compounds added to the tubing (χ^2^ = 15.75, df = 3, *p* = 0.001), with significant differences between the control and ethyl anthranilate (Tukey’s test, *p* = 0.006) and between the control and p-anisaldehyde (Tukey’s test, *p* = 0.005). In contrast, the number of ants did not differ significantly in any other pairwise comparison (*p* > 0.05). The highest number of ants was recorded in the controls (2.92 ± 2.08 ants per emitter ± SE); in the tubes with repellents, the number of ants per emitter ranged between 0.3 (EA) and 1.03 (CEO) (Figure 8A). 

The proportion of damaged diaphragms was also significantly different among treatments (χ^2^ = 42.81, df = 3, *p* < 0.001), being higher in controls than in any other case (Tukey’s test, *p* < 0.001). No significant differences in damage were observed among the diaphragms of the tubes with repellents (EA vs. ANI: *p* = 0.983; CEO vs. EA: *p* = 0.983; CEO vs. ANI: *p* = 1). Almost 60% of the observed diaphragms in controls were damaged (59.26 ± 35.74% of damaged diaphragms ± SE), while those in tubes with ANI and CEO showed much lower values (3.33 ± 1.92), and no damage at all was observed in the tubing with EA (Figure 8B). 

**Figure 9:**
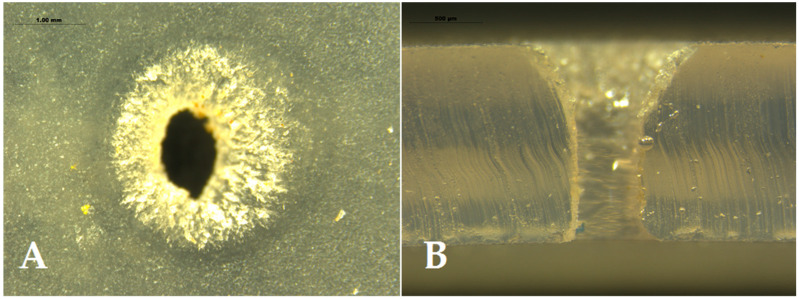
Some examples of the level of damage observed in the silicon diaphragms of emitters belonging to the control tubing (Block 2). (**A**) Upper view of a damaged diaphragm, showing prominent erosion marks around the inner orifice; (**B**) lateral view of the transversal cut of another emitter, with a deep “truncated cone”.

Similarly, the volume of the eroded diaphragm also differed among the four types of tubing (χ^2^ = 27.03, df = 3, *p* < 0.001). Again, controls showed significantly higher damage than the tubing with repellents (Tukey’s test, *p* < 0.001), with a substantial mean volume of missing silicone (0.13 ± 0.08 mm^3^ missing per emitter ± SE) (Figure 8C and Figure 9). The treatments with repellent compounds did not show statistical differences among each other (*p* > 0.05), with ANI exhibiting slightly higher damage (0.003 ± 0.002 mm^3^ missing per emitter ± SE). 

## 4. Discussion

Despite the limited attention received in the scientific literature, ant damage to microirrigation equipment is well known in agriculture because of the substantial economic losses it implies. The present study supports the incorporation of repellent substances into drip irrigation tubing as an efficient alternative to reduce this damage. In the first part of the study, via olfactory trials, we selected different substances that exhibited significant repellence against ants, both in liquid form and after being incorporated into polyethylene tubing. This was undertaken because some of their properties, including repellence, could be expected to be lost when added to plastic. Cinnamon essential oil, p-anisaldehyde and citronella essential oil offered the best results in liquid form, and ethyl anthranilate (EA) also showed satisfying results in plastic. Therefore, EA, cinnamon essential oil and p-anisaldehyde were selected for field trials, where they led to significantly reduced damage in comparison with tubing without repellents. This suggests not only the repellence of these substances but also their potential stability in plastic as the experimental tubing was exposed for three months to the degradation processes that occur naturally in the soil.

Numerically, EA offered the best results, with no damaged emitters in tubing with this compound. The use of EA as a repellent was initially suggested by Kain et al. [31] after an extensive search for new insect repellents through bioinformatics and structural analyses. Since then, several studies demonstrated the high repellence of this compound against different types of insects, including ants [32,33,56], which agrees with our results. EA is nontoxic, rated safe for human use and inexpensive [57]. Together with our results, EA seems to be an excellent candidate for the protection of irrigation systems. Nevertheless, p-anisaldehyde and cinnamon essential oil showed very similar results, and they are also natural and nontoxic at low doses to humans and the environment [36,37]. This suggests that the final choice among the substances for the commercial implementation of this method would be based on economic and/or logistical criteria established by manufacturers. 

Before this work, the scarce attempts to use repellent incorporation to protect microirrigation systems against animal agents were unsuccessful, using mostly toxic compounds exhibiting efficiency that was also short-term due to the surface-spraying method employed [16,27,29]. Unlike those trials, our methodology exhibited a successful protective effect, which could be attributed to two main factors. We first tested the repellent effect against ants of the substances incorporated into polyethylene tubing. Then, we used more complex processes for the incorporation of these substances into the tubing, including an initial compounding and a subsequent extrusion and/or injection into the different tubing parts of the masterbatches. These processes aimed to reduce thermal degradation and extend the durability of the repellent effect, which, as mentioned above, was achieved in our study. To our knowledge, this is the first time that this technology has been tested for protecting irrigation pipes, while in other structures, such as power cables and gas pipelines, similar attempts have been previously conducted against rodents, showing promising results [58,59,60].

In addition to efficiency and durability, another objective of repellent incorporation as a control method is environmental safety, which is why we only selected natural substances generally regarded as nontoxic at low doses. Repellence should not be associated with toxicity; indeed, by definition, a repellent is simply something that causes insects to make oriented movements away from its source [61,62]. Toxicity was especially undesirable in our study since it could result in water and soil contamination and subsequently harm the wild flora and fauna. The damaging potential of ants on irrigation pipes made them target organisms for our research, but they also play a key role in the soil as ecosystem engineers [63,64], and thus any toxic effect of the substances employed could have unwanted effects on the agroecosystem. The same applies to other potentially harmful agents to irrigation systems, which may also be beneficial to arable crops through ecosystem services, such as pest control or the removal of weed seeds [65,66,67]. As mentioned above, all repellents considered in our study are generally regarded as nontoxic to humans and the environment at low doses and therefore are not expected to have any detrimental effect on the ecosystems where the irrigation systems are installed. However, since we did not include any assessment of their effect on the local soil organisms and underground water, further studies in this line would be welcome to ensure the environmental protection of our method.

In our study area, ants seemed to be the main threat to irrigation systems. Growers have not reported any damage associated with rodents or other large agents, and our tubes only exhibited the presence of ants and internal damage. This could be partly explained by the fact that we reproduced only subsurface conditions, considering the relevance of SDI in the Mediterranean Basin due to the efficient water use, reduced energy costs and protection from weather conditions that this system ensures [68,69]. It is known that SDI systems in other areas may be strongly damaged by rodents [29,70], while surface systems such as microsprinklers or SD are vulnerable to birds and different chewing mammals [71,72,73]. Therefore, new tests in other areas and/or using surface systems would be highly advisable to determine whether our method can simultaneously control ants and larger agents, which is one of its main potential advantages compared to traditional approaches. 

The six ant species detected in the emitters had their small sizes in common. All individuals showed maximum body widths of less than 0.7 mm (the authors´ observations), and the maximum width of most of these species is known to rarely exceed 0.5 mm [52,74,75]. This underlines body size as a key factor in the damage-causing potential of ants. Other factors, however, are also expected to affect the ability of ants to harm irrigation systems and may be investigated for an optimal application of our findings. For example, Chang and Ota [16] noticed a direct relationship between ant damage and depth burial and observed that the damage increased with the content of organic matter, CO_2_, and especially water, inside the tubing. Water or moisture content is believed to be the main factor attracting ants to irrigation pipes when not in use, especially in arid environments [18,22,26]. In our study, we obtained satisfying results even when tubes were filled with honeydew, which is expected to increase the attractiveness to ants and could even disrupt the effect of the repellent substances. Thus, we could expect even higher efficiency under normal conditions with tubes only containing water or moisture, but this needs to be checked in future research. In summary, the installation of our systems at shallow depths (whenever possible) and with proper equipment maintenance and the avoidance of both extended periods of system non-use and the accumulation of organic matter seems to be an advisable strategy for contributing to protection against ants. 

## 5. Conclusions

The present research represents the first attempt to develop a microirrigation system that protects against ants exclusively based on the incorporation of repellent substances. The method we proposed was based on a careful selection of nontoxic compounds with a proven repellent effect on ants and the use of complex processes, such as injection and extrusion, to incorporate these substances into polyethylene tubing. We observed extremely low damage in the repellent tubes when compared to control tubes, which showed clear evidence of ant-caused damage after a 3-month exposure period in the field. This suggests that the proposed methodology may be an excellent option for the protection of microirrigation systems because of its efficiency, durability and the null effects that it is expected to have on the environment. Since many other animal agents are known to be able to harm this type of structure, further studies in other areas and/or focused on other damaging agents are strongly recommended to assess the overall degree of protection provided by this method. Other research lines focused, for example, on the durability of the repellent effect in the field or the potential effects of repellents on soil organisms and water will also help to optimize the implementation of this method prior to commercialization.

## Figures and Tables

**Table 1:** Statistics for chi-square goodness-of-fit tests for the effect of the volatiles of different compounds (CEO: cinnamon essential oil; CIT: citronella essential oil; ANI: p-anisaldehyde; EA: ethyl anthranilate; PER: permethrin; EUC: eucalyptol) on the olfactory response of Pheidole pallidula. χ2 = chi-square values; df = degrees of freedom. Compounds that showed significant repellence are typed in bold in each comparison.

Type of Test	Comparison	χ^2^	Df	*p*
Compounds vs. Control (Liquid)	**CEO** vs. Control	16.90	1	<0.001
	**CIT** vs. Control	6.40	1	0.001
	**ANI** vs. Control	2.51	1	0.025
	EA vs. Control	1.60	1	0.073
	PER vs. Control	1.60	1	0.073
	EUC vs. Control	0.10	1	0.752
Pairwise comparisons among compounds (Liquid)	**ANI** vs. PER	19.60	1	<0.001
	**CEO** vs. PER	10.76	1	<0.001
	**ANI** vs. CEO	8.11	1	<0.001
	**CIT** vs. PER	6.40	1	0.001
	**EA** vs. PER	2.51	1	0.025
	CEO vs. EA	1.60	1	0.206
	ANI vs. EA	0.90	1	0.343
	CIT vs. CEO	0.40	1	0.527
	CIT vs. EA	0.40	1	0.527
	CIT vs. ANI	0.10	1	0.752
Compounds vs. Control (LDPE 5%)	**CEO** vs. Control	4.92	1	0.002
	ANI vs. Control	1.60	1	0.206
	EA vs. Control	0.90	1	0.343
	CIT vs. Control	0.90	1	0.343
Compounds vs. Control (HDPE 3%)	**ANI** vs. Control	14.40	1	<0.001
	**CEO** vs. Control	4.92	1	0.002
	**CIT** vs. Control	2.51	1	0.025
	EA vs. Control	0	1	1
Compounds vs. Control (HDPE 5%)	**CEO** vs. Control	8.11	1	<0.001
	**EA** vs. Control	4.92	1	0.002
	**ANI** vs. Control	4.92	1	0.002
	**CIT** vs. Control	3.60	1	0.010

## Data Availability

Data is available through the authors.

## Supplementary Materials

The following are available online at www.mdpi.com/article/10.3390/insects13040395/s1, Table S1: List of compounds selected as potential repellents to be incorporated to polyethylene SDI tubes, including information on their main features and their reported effects on ants.
