# Supplementary material for: Natural Repellents as a Method of Preventing Ant Damage to Microirrigation Systems
Source: Insects. 2022 Apr 18;13(4):395. doi: 10.3390/insects13040395 (PMC9028605; doi:10.3390/insects13040395)
Supplement: Supplementary file 1 [file insects-13-00395-s001.zip › insects-1658626-supplementary.pdf]

Table S1. List of compounds selected as potential repellents to be incorporated into polyethylene SDI tubes, including information on their main features and their reported effect on ants.

Compound	Features	Effect on ants/other insects	References
**Ethyl anthranilate**	ABIR (anthranilate-based insect repellent); cheap, nontoxic and widely used as an additive in food and cosmetics industries	Demonstrated promising repellent activity against head lice and different species of mosquitoes. In extremely low concentrations, it also prevented red imported fire ant (RIFA: *Solenopsis invicta* Buren) nesting	Kain et al. [31]<br>Islam et al. [32]<br>Chen et al. [33]
**Eucalyptol (1,8-cineol)**	Main constituent (ca. 90%) of the eucalyptus essential oil; aromatic and medicinal properties	Showed a complete repellent effect on RIFA feeding activity, preventing attacks on *Tenebrio molitor* L. larvae sprayed with eucalyptus oil and reducing climbing and feeding activity in sprayed ants	Wang et al. [34]
**p-anisaldehyde (4-methoxybenzaldehyde)**	Floral volatile present in different plant species; main component of the floral bouquet of male flowers of *Petasites fragans*	Exhibited repellent effect on *Formica aquilonia* (Yarrow) in olfactory tests. Strongly attractive to pollinators	Theis et al. [35]<br>Pattrick et al. [36]
**Cinnamon essential oil**	Extracted from the bark or the leaves of different *Cinnamomum* species; commonly used in food and chemical industries due to its aroma	Both essential oil and leaves of indigenous cinnamon (*C. osmophloemum*) showed an excellent inhibitory effect that controls RIFA	Cheng et al. [37]<br>Huang et al. [38]
**Citronella essential oil**	Extracted from the Poaceae *Cymbopogon nardus* and *Cymbopogon winterianus,* few toxic to mammals and extensively commercialized as insect repellent	Prevented movement of RIFA and the Argentine ant (*Linepithema humile* (Mayr)); in high doses, it caused significant ant mortality of both species	Wiltz et al. [39]
**Permethrin**	Odorless and biodegradable synthetic pyrethroid insecticide, derived from the plant *Chrysanthemum cinerariifolium*	At very low doses, it caused aversion in a great variety of arthropods	Islam et al. [42]
